# The future of stroke care: how telestroke is bridging the gap in stroke care

**DOI:** 10.1007/s11239-025-03150-x

**Published:** 2025-07-09

**Authors:** Lucio D’Anna, Marilena Mangiardi, Francesco Corea, Christoph Gumbinger, Rene Handschu, Olfa Kaaouana, Vasileios Tentolouris-Piperas, Laetitia Yperzeele, Vaso Zisimopoulou

**Affiliations:** 1 Department of Stroke and Neuroscience, Charing Cross Hospital, Imperial College London NHS Healthcare Trust, London, UK; 2 Department of Brain Sciences, Imperial College London, London, UK; 3 Department of Neurology, Inselspital, Bern University, Bern, Switzerland; 4 Stroke Unit Division, San Camillo Forlanini Hospital, Rome, Italy; 5 Stroke & Neurology Units Ospedale San Giovanni Battista, Foligno, Italy; 6 Department of Neurology, Heidelberg University Hospital, Heidelberg, Germany; 7 Department of Neurology, Schweinfurt, Germany; 8 Department of Neurology, Jean Bernard Hospital Valenciennes, Valenciennes, France; 9 Department of Neurology, Eginitio University Hospital, University of Athens, Athens, Greece; 10 Stroke Unit and NeuroVascular Center, Dept. of Neurology, Antwerp University Hospital, Edegem, Belgium; 11 Translational Neurosciences Research Group, Faculty of Medicine and Health Sciences, University of Antwerp, Antwerp, Belgium; 12 Department of Neurology, Stroke Unit Division, Athens Euroclinic Hospital, Athens, Greece; 13 Department of Brain Sciences, Imperial College London, London, UK

**Keywords:** Stroke care, Telestroke, Telemedicine

In recent years, telestroke technology has emerged as a cornerstone for delivering high-quality stroke care in remote and underserved regions across the globe. While this editorial draws on experience and data from Europe, many of the challenges and opportunities discussed are shared across international healthcare systems [1]. This innovative healthcare solution connects patients with specialized neurologists in real time, thereby facilitating accurate diagnosis and timely intervention with a focus on the critical early stages of stroke care. Given that stroke is a time-sensitive condition where every minute counts, telestroke represents a pivotal tool for reducing treatment delays, morbidity, and mortality [2].

This editorial explores the potential of telemedicine to bridge gaps in stroke care and argues for its initial application to remain focused exclusively on stroke rather than broader neurological conditions.

## The urgency of timely stroke intervention

Stroke remains one of the leading causes of death and disability worldwide, with ischemic strokes accounting for the majority of cases [3, 4]. Immediate treatment, including thrombolysis or mechanical thrombectomy, is essential for maximizing recovery, as neuronal death begins within minutes of oxygen deprivation [5]. Moreover, for hemorrhagic stroke, evidence highlights the time-critical nature of interventions such as aggressive blood pressure lowering and anticoagulation reversal, with earlier initiation consistently linked to better outcomes, as supported by major guidelines and trials [6–8]. However, not all hospitals even if they treat stroke patients have the resources to provide specialized stroke care on-site, particularly in rural or low-resource settings. Compounding this challenge, many countries face the dual pressures of aging populations and expanding eligibility for acute stroke treatments, which are expected to further strain healthcare systems worldwide [9]. Current neurologist-to-population ratios—averaging 9.4 per 100,000 in Europe—exacerbate the difficulty of ensuring timely and equitable access to care [10]. The scarcity of stroke-specialized neurologists intensifies the challenge, particularly in rural hospitals. Adding to the strain, many people are relocating from urban centers to rural or suburban regions, driven by lifestyle changes, remote work opportunities, and affordable housing. This growing shift toward rural living is increasing the demand for accessible healthcare in areas often lacking dedicated stroke specialists, putting rural hospitals under even greater pressure to provide critical stroke care in the crucial minutes after onset.

In this landscape, Telestroke has demonstrated its capacity to address these disparities [11]. By connecting local care providers with stroke neurologists via video consultations, telestroke enables evidence-based decision-making in facilities lacking on-site expertise. The exclusive focus of telestroke on stroke care could ensure that resources are directed toward refining and optimizing care protocols that cater specifically to stroke patients.

By narrowing the scope, telestroke networks can develop specialized workflows, invest in targeted training for local staff, and maintain high standards in stroke diagnosis and treatment.

In an era marked by a rising absolute number of stroke cases due to population aging, persistent shortages of stroke specialists in many underserved areas, and changing population dynamics including movement toward suburban and semi-rural regions, telestroke stands as a crucial, high-impact tool [12]. Its targeted approach minimizes the risk of delays or misdiagnosis, providing healthcare systems with an essential means to deliver life-saving care to patients in need, no matter where they live.

## Telestroke as an equalizer in stroke care access

One of the most compelling benefits of telestroke is its ability to bridge geographic and socioeconomic gaps in healthcare access [1]. Rural hospitals, which often lack on-site stroke neurologists, can now provide high-quality stroke care through telestroke networks. Remote consultations with stroke neurologists allow these hospitals to administer thrombolytic therapy or decide on patient transfer quickly, giving patients in rural or underserved areas a fighting chance at recovery. Numerous studies underscore the effectiveness of telestroke in reducing treatment delays and improving patient outcomes [13]. For instance, hospitals with telestroke capabilities report significantly higher rates of thrombolysis administration and faster response times [12, 14]. This focused, stroke-specific model allows for a streamlined process in rural and underserved settings, transforming what would otherwise be logistical challenges into life-saving interventions. Telestroke’s narrow concentration enhances these benefits, enabling rural providers to establish consistent, high-quality stroke care protocols without the distractions and resource strains of addressing non-stroke emergencies [12].

## Leveraging technology for stroke-specific care

Telestroke leverages state-of-the-art technologies that have advanced stroke diagnostics and treatment options. Through real-time imaging and teleconsultations, neurologists can remotely review brain scans, assess the type and location of a stroke, and recommend treatment options promptly [15]. Artificial intelligence (AI) now aids in interpreting imaging results, detecting clots or bleeding, and assessing stroke severity, thereby making it easier to provide timely and precise interventions. New tools like wearable devices add a layer of continuous monitoring for stroke patients, allowing providers to remotely track vital signs and respond to any concerning changes. Blockchain technology is also being explored for secure data sharing within telestroke networks, ensuring rapid and private access to critical patient information. Additionally, virtual reality (VR) offers immersive training for local providers and engaging rehabilitation options for patients, enhancing recovery experiences [16]. A bespoke stroke telemedical service enables healthcare systems to concentrate investments in these cutting-edge technologies specifically for stroke care. While some have suggested expanding telestroke into a broader teleneurology model to enhance sustainability, particularly in resource-limited rural hospitals, this approach requires careful consideration. Broader use may support local care delivery and financial viability; however, it is essential that the core elements of rapid response, stroke-specific protocols, and time-critical decision-making remain intact. A stroke-focused model ensures that the precision, efficiency, and quality of acute stroke care are not compromised [17].Future research comparing stroke-specific and integrated teleneurology models would help clarify the optimal balance between sustainability and specialized care.

## Improved outcomes and patient experience

Patient outcomes have consistently shown improvement with telestroke services, with multiple studies reporting shorter time-to-treatment, higher thrombolysis rates, and better functional outcomes compared to conventional care models [14, 17]. Studies highlight that patients who receive care through telestroke networks have a shorter time-to-treatment and better functional outcomes, with many avoiding severe disabilities. Hospitals utilizing telestroke experience fewer complications and readmissions, reflecting the benefit of specialized, stroke-centered care protocols. Moreover, telestroke provides reassurance to patients and families, particularly in regions where specialized stroke care would otherwise be unavailable. The immediacy and expertise offered through telestroke foster trust in the healthcare system, empowering patients and their loved ones during an emotionally challenging time. This patient-centric focus on stroke improves both clinical outcomes and the overall healthcare experience.

## Policy support and the path forward

For telestroke to maximize its impact, policy support and infrastructure investment are critical. Governments and healthcare systems should prioritize telestroke as a focused, stroke-specific service, allocating resources to expand this technology’s reach to more rural and underserved communities. Telemedicine reimbursement policies should be adapted to support telestroke networks, ensuring that providers are compensated for teleconsultations and that essential equipment is available and maintained. Investment in infrastructure, particularly in rural hospitals, is vital to telestroke’s success [18]. Providing training to local healthcare staff, equipping facilities with necessary technology, and ensuring consistent connectivity are essential to make telestroke a reliable, high-quality service. Policy initiatives should also aim to integrate telestroke networks into national healthcare strategies, recognizing telestroke’s role in reducing stroke-related mortality and disability and improving equitable access to care.

## Global and european adoption of telestroke: evidence and obstacles

Despite the well-documented benefits of telestroke, its adoption across Europe and other regions remains variable. Some countries, such as Germany and Finland, have developed well-integrated telestroke networks, while others are still in early stages of implementation or operate on a limited scale. In contrast, the United States has seen broader adoption, supported by national initiatives and reimbursement frameworks. Quality data from established networks show clear improvements in door-to-needle times, thrombolysis rates, and patient outcomes, reinforcing the clinical value of telestroke systems [11, 14]. However, barriers to widespread implementation persist. These include uneven digital infrastructure, limited funding for equipment and staff training, insufficient reimbursement for teleconsultations, and legal or regulatory uncertainties related to cross-border care [2, 12]. Addressing these challenges through targeted policy support and investment is essential to ensure equitable and sustained access to high-quality telestroke services across Europe and beyond.

## Conclusion

### The future of telestroke as a focused stroke care solution

Telestroke is a powerful innovation in modern healthcare, offering a focused solution that saves lives by bridging gaps in access to stroke treatment. By dedicating telestroke exclusively to stroke intervention, healthcare systems can harness the technology’s full potential, refining workflows, enhancing staff training, and maintaining the highest standards in stroke-specific diagnostics and treatment. Expanding telestroke beyond stroke care risks diluting these benefits, potentially compromising the quality of care in urgent, time-sensitive situations. Looking ahead, the future of telestroke lies in its commitment to specialized, focused care. With the support of policymakers, healthcare providers, and continued technological advancement, telestroke can remain a transformative force in stroke care, ensuring that patients everywhere receive the life-saving treatment they need, when they need it. As a specialized model, telestroke does not merely elevate the standard of care; it fundamentally revolutionizes and transforms healthcare delivery itself, bringing it closer to achieving true equity in medical access and outcomes. Although this editorial focuses on the European experience, the lessons learned from telestroke implementation are highly relevant to global stroke care. Across diverse healthcare systems—from North America to Asia and low- and middle-income countries—telestroke presents a scalable and impactful model for improving timely access to expert stroke care.
